# Malnutrition among 3 to 5 Years Old Children in Baghdad City, Iraq: A Cross-sectional Study

**DOI:** 10.3329/jhpn.v31i3.16827

**Published:** 2013-09

**Authors:** Hasanain Faisal Ghazi, Jamsiah Mustafa, Syed Aljunid, Zaleha Md. Isa, Mohammed A. Abdalqader

**Affiliations:** ^1^Department of Community Health, Universiti Kebangsaan Malaysia Medical Centre, Kuala Lumpur, Malaysia; ^2^United Nations University, International Institute for Global Health, Kuala Lumpur, Malaysia

**Keywords:** Children, Insecure living environment, Underweight, Iraq

## Abstract

The unstable geopolitical situation in Iraq since 2003 still affects the health of people, especially children. Several factors may indirectly affect a child's nutritional status. The main aim of this study was to identify factors contributing to malnutrition among 3 to 5 years old children in Baghdad city, Iraq. Two hundred twenty children aged 3 to 5 years were chosen randomly from four kindergartens in Baghdad city according to the cross-sectional design. The nutritional status of the children was assessed using a weight-for-age z-score based on the World Health Organization 2007 cutoff points, in which any child with a z-score of <-2 is considered to be malnourished. The overall prevalence rate of underweight children was 18.2%. There was no significant difference in the prevalence rate between males and females (p=0.797). However, the percentage of underweight children was slightly higher among females (18.9%) compared to males (17.6%). There was no association between parents’ educational level or employment status and childhood malnutrition. There was no association between a family's movement from their house and childhood malnutrition (p=0.322). Living in an unsafe neighbourhood and having a family member killed during the past five years were significantly associated with childhood malnutrition (p=0.016 and 0.018 respectively). Childhood malnutrition is still a public-health concern in Baghdad city, especially after the war of 2003. Malnutrition is significantly associated with living in unsafe neighbourhoods and at least one family member having been killed during the past five years.

## INTRODUCTION

Sociodemographic factors, such as age, sex, family-size, and number of children may indirectly contribute to a child's nutritional status and affect child health. The complex aetiology of childhood malnutrition is a multifactorial process and related to many socioeconomic and sociodemographic factors.

The primary determinants of malnutrition were unsatisfactory food intake, severe and repeated infections, or a combination of the two. The nutritional status of children may also be affected by socioeconomic and demographic factors, such as paternal and maternal occupation and education, marital status, family income, nutritional knowledge of mothers, location of house (urban or rural), gender, and water supply (1).

The sex of children is an important influential factor in determining nutritional status. Some subnational studies on gender differences in anthropometric status found female children in India to be at disadvantage (2). Some have found no gender differences (3), and a few have found male children to be more often stunted or underweight (4,5).

The family-size and number of children living in the same house are important factors for nutritional status of the children, which reflects the quality of care given to those children. A study done to assess the nutritional status of children aged 6 to 59 months in Livingstone, Zambia, in 2005, found that 43% of undernourished children were associated with extended families (five to seven members) while 36% were associated with nuclear families (two to four members) (6).

One important factor relating to childhood nutrition is the mother's education. Many studies have demonstrated that improvements in secondary school enrollment rates among females are estimated to be responsible for 43% of the total 15.5% decline in the childhood underweight rate in developing countries during the period 1970–1995 (7). The father's education also emerged as an important factor that was significantly associated with underweight status among under-five children. Analysis showed that children whose fathers had higher education had lower levels of weight deficiency than those with non-literate fathers. Usually, the father is the main earner and decision-maker in a family; so, their higher education plays an important role in ensuring better nutritional status of children (8).

Environmental factors play an important role in child's nutritional status, especially in Iraq. Thirteen years of economic sanctions followed by the 2003 war and eight years of unstable socioeconomic and political security, especially in Baghdad city, have affected the daily life of Iraqi families and children, especially in respect of their nutritional status. Nutritional status is the result of a complex interaction between the food we eat, our overall health, and the environment in which we live. Food, health, and caring are the three “pillars of well-being” (9).

Basic services, such as electricity and water supply, have been disrupted, and a rise in food prices has affected food security at the household level (10). This will, in turn, cause malnutrition among family members, especially the children.

The unstable living situation in Iraq since 2003 still affects the physical and mental health of the people. Violence dominates everyday life of the Iraqis. As car bombings, roadside bombings, suicide bombings, murders, sniper attacks, kidnappings, drive-by shootings, torture, and sectarian killings have become daily events in many cities (11).

Gabriela Guerrero-Serdan (3) carried out a study in Iraq to assess the effects of war on nutrition and health. He assumed that war has affected the physical growth of children whereby those born after the war in high-intensity conflict areas had lower height-for-age z-scores than those born in less violent areas. He also found that weight-for-age z-scores increased in 2004 but decreased in 2006. These results suggest that children are not losing weight but rather are not growing properly or failing to thrive (FTT) in high-violence areas.

The main objective of this cross-sectional study was to assess the prevalence of malnutrition among children aged 3 to 5 years in Baghdad city and to determine the associated risk factors, such as sociodemographics and living environment.

## MATERIALS AND METHODS

A cross-sectional design was used; the sample-size was 220 aged between 3 and 5 years selected randomly from four different kindergartens in Baghdad city in May 2009. A list of 144 kindergartens in Baghdad was obtained from the Ministry of Education, and then four kindergartens were selected from different areas of the city, representing both high and low socioeconomic classes. Subsequently, a complete list of students’ names in each selected kindergarten was obtained, and 55 children from each kindergarten were then identified by simple random sampling method.

Calculation was done using Kish's formula (1965) (12) as follows:

N=(Zα/2)^2^ (P) (1-P)/D^2^

where Zα/2 is the standard normal value at 95% CI=1.96; N is the sample-size, D (delta) is the precision of 5%, or the marginal error (in this study, we take it as 0.05).

With an expected prevalence of 13.2% based on a previous survey in Baghdad (11), confidence interval of 95%, and Delta value equal to 0.05,

N=(1.96)^2^ (0.132) (1-0.132)/(0.05)^2^=176

Thus, the minimum sample-size calculated was 176. However, 25% (44 respondents) were added because of the non-response rate. Therefore, the final sample-size was 220 respondents.

Both questionnaire responses and anthropometric measurements were used in this study. A self-administered questionnaire was distributed to respondents’ parents after getting their approval for participation in the study (child assent).

A well-trained co-researcher collected data. Weight was measured using a digital weighing machine (Beurer) manufactured in Germany. Children were weighed after taking off their shoes and with minimum possible clothing. Then, their weight-for-age z-score was obtained and compared with the WHO cutoff points.

Malnutrition in this study was assessed by underweight status or low weight-for-age z-score as an indicator of childhood malnutrition. Underweight is 2 z-score below the international reference for weight-for-age. The z-scores were calculated for a child's weight, given age and gender, by subtracting the median weight in the reference population and dividing by the standard deviation of the reference population (13).

Parents’ educational level is defined in this study as either low (ranging from non-literacy to primary education only) or high (secondary school, generally the final stage of compulsory education, and/or university degree).

An insecure living environment is defined in this study as lacking a sense of security or affording no ease or re-assurance, in which life is threatened. This was evaluated according to whether (i) the family had been forced to leave their home, (ii) one of the family members has been killed, or (iii) the living area was felt by the respondents to be insecure.

The questionnaire was tested by choosing 15 parents to answer, to ensure that it was easily understandable. Most of the parents found that the questionnaire was good, straightforward, and easy to understand. The result of this pre-test was used for improving the phrasing of the questions.

The study was approved by the Research and Ethics Committee of the Universiti Kebangsaan Malaysia Medical Centre.

### Statistical analysis

The association between underweight and sociodemographic factors and living environment variables was examined by a chi-square test (for categorical variables). Data analysis was carried out using Statistical Package for Social Sciences (SPSS) (version 16.0); Epi Info (version 6.0) was used for obtaining the weight-for-age z-score.

## RESULTS

The prevalence of malnutrition in this study was 18.2%. The mean age of children was 3.92±0.8 years. The average body-weight of the respondents was 15.63±3.18 kg as shown in Table 1.

The minimum number of family members was two, and the maximum number was eight, with an average of 4.63. The minimum number of children within a family was one child, and the maximum number was six.

More than 90% of the respondents’ parents were still married. The most common level of education in mothers was high at 70%, with 30% educated to a low level. Among fathers, 88.6% were educated to a high level, with 11.4% educated to a low level. The highest rate of malnourishment in children was found in the low-education group (24%) while, in the high-education group, it was 17.4%. Almost 96% of the fathers were working and only 4% not working. Among the mothers, 55% were working, and 45% were not working. The largest percentage of malnourishment was among the children of working mothers (19.4%).

Table 2 shows the relationship between sociodemographic factors and the nutritional status of children. There was no significant difference (p=0.797) between males and females regarding malnutrition, although the level of underweight was higher in females (18.9%) than in males (17.6%). Marital status (p=0.113), mother's education (p=0.252), father's education (p=0.423), mother's working status (p=0.678), and father's working status (p=0.748) showed no significant association with children's nutritional status.

Table 3 shows the relationship between insecure living environment and child's nutritional status where there was a significant relationship between families with a member killed in the past five years of violence, with p=0.018 and prevalence odds ratio (POR) of 2.4, which means that families in which any member was killed had 2.4 times greater chance of having malnourished children. The relationship between living in an insecure area and child's nutritional status was also significant, with a p value of 0.016 and prevalence odds ratio of 2.3 while a family moving from their house because of violence was not significantly associated with child's nutritional status.

**Table 1.** Anthropometric measurements (weight and weight-for-age z-score)

Variable	Mean	SD	Minimum	Maximum
Age (years)	3.92	0.8	3	5
Weight (kg)	15.36	3.18	9.20	25.1
Weight-for-age z-score	-0.538	1.371	-3.86	3.02

## DISCUSSION

There was no significant difference in the prevalence of malnutrition between male and female respondents (p=0.797). However, the level of underweight was higher among females (18.9%) than among males (17.6%). The findings of this study are supported by other studies done in Oman, Eastern Nepal, and Ethiopia (14-16). However, according to Gabriela (3), there is no difference in weight-for-age or weight-for-height between boys and girls in Iraq, and this reflects the fact that girls in Iraq are more vulnerable or have a greater possibility of reaching potential linear growth. This could suggest that girls in Iraq are biologically stronger at an early age.

**Table 2.** Relationship between sociodemographic factors and child's nutritional status

Variable	Nutritional status of children			POR	95% Confidence interval
	Underweight N (%)	Normal N (%)	p value^a^		
Child's sex					
Male	22(17.6)	103(82.4)	0.797	0.914	0.459-1.820
Female	18(18.9)	77(81.1)			
Parent's marital status					
Still married	34(16.9)	167(83.1)	0.113	0.441	0.157-1.242
Separated or divorced	6(31.6)	13(68.4)			
Mother's education					
Low	15(22.7)	51(77.3)	0.252	1.518	0.741-3.110
High	25(16.2)	129(83.8)			
Father's education					
Low	6(24.0)	19(76.0)	0.423	1.495	0.556-4.023
High	34(17.4)	161(82.6)			
Mother is working					
Yes	19(19.4)	79(80.6)	0.678	1.157	0.582-2.299
No	21(17.2)	101(82.8)			
Father is working					
Yes	38(18.0)	173(82.0)	0.748	0.769	0.154-3.847
No	2(22.2)	7(77.8)			

^a^Pearson chi-square test was performed;

POR=Prevalence odds ratio

**Table 3.** Relationship between insecure living environment factors and child's nutritional status

Variable	Nutritional status of children			POR	95% Confidence interval
	Underweight N (%)	Normal N (%)	p value a		
Family members moved from their house					
Yes	21 (21.0)	79 (79.0)	0.322	1.413	0.711-2.808
No	19 (15.8)	101 (84.2)			
Family member(s) killed in the last 5 years					
Yes	15 (29.4)	36 (70.6)	0.018*	2.400	1.149-5.015
No	25 (14.8)	144 (85.2)			
Is living area secure?					
No	22 (26.2)	62 (73.8)	0.016*	2.326	1.161-4.659
Yes	18 (13.2)	118 (86.8)			

^a^Pearson chi-square test was performed;

*Level of significance p<0.05;

POR=Prevalence odds ratio

In this study, the most common level of education among both mothers and fathers was high (70.0% and 88.6% respectively). The low educational level ranged from non-literacy to primary education while the highly-educated group attended secondary schools and universities. The prevalence of underweight was higher among children of low-educated mothers (22.7%) and low-educated fathers (24.0%) compared to those of more highly-educated parents. The association between parents’ educational level and childhood malnutrition, however, was not significant (p=0.252 for the mother's education and p=0.423 for the father's education). Popkin (17) and Aaby *et al*. (18) revealed that mothers of the most malnourished children worked outside their homes.

The unstable living environment in Baghdad in the past eight years affects family and child health. According to UNICEF, the war in March 2003 caused nearly 15% of Iraq's total population to flee from their homes. About 2.2 million people have fled to neighbouring countries, mostly Jordan and Syria. Another 1.9 million are displaced within Iraq, and half of the nearly 4 million displaced Iraqis are children (19).

In this study, the percentage of families that had moved from their homes because of violence was 45%. This could have affected children's health as they stayed in camps or relatives’ houses where sanitation may not have been good. Of the respondents’ parents, 38% said that their living area was not secure while 23% of families had one member killed in the violence, leaving either father or mother with the sole responsibility for providing food and supplies to the family.

The association between an insecure living area, a family member killed and childhood malnutrition was significant. This result is supported by other studies, such as by Aaby *et al*. (18). Their research following the conflict in Guinea-Bissau found that residents in a non-camp setting were more malnourished and had higher mortality rates than the refugees. Major improvements in nutritional status and a decline in mortality were found among children of residents and refugees as soon as they returned home, despite the fact there was no improvement in food availability.

Aldoori *et al*. (20) concluded in their study in Basrah city in southern Iraq following the 1991 war that 24% of children were stunted. Stunting and low weight-for-age were significantly higher among children from families with low socioeconomic status. Comparison of these data with an earlier nutritional survey in the area showed that the nutritional status of children in Basrah city had worsened because of successive armed conflicts.

### Limitations

There were some limitations in this study. First of all, its cross-sectional design only measured the prevalence at specific point of time in specified areas. Second, the respondents were selected from kindergartens and not from a community because of the unstable security situation in Baghdad that made that type of survey difficult. The third limitation is the recall bias of the respondents’ parents, especially in their evaluation of the security situation in their living areas. Furthermore, family monthly income was not included in this study due to issues of under- and over-reporting.

### Conclusions

Childhood malnutrition is still a major public-health problem in Baghdad. This study showed that malnutrition is significantly related to insecure living areas and at least one family member having been killed in the past five years.

### Recommendations

The study recommends that the nutritional know-ledge of parents, especially mothers, be increased. The health ministry and other relevant ministries should try various methods of disseminating this knowledge to members in the community, for example, through media, workshops, etc.

## ACKNOWLEDGEMENTS

This study was funded by the UKM Medical Centre Fundamental Research Grant (Code number FF-092-2009), without which it would not have been possible.
